# Someone Like Me? Disability Identity and Representation Perceptions

**DOI:** 10.1007/s11109-024-09969-z

**Published:** 2024-08-28

**Authors:** Stefanie Reher, Elizabeth Evans

**Affiliations:** 1 University of Strathclyde, Glasgow, Scotland; 2 University of Southampton, Southampton, England

**Keywords:** Political representation, Disability, Identity, Stereotypes, Survey experiment

## Abstract

Studies have shown that citizens from minoritized groups, including women and people of color, tend to feel better represented by politicians who share their identity, often translating into electoral support. Is this also the case for disabled people, one of the largest yet often ignored minority groups in our societies? Analyses of data from a conjoint survey experiment with 6,000 respondents in the UK and US show that disabled people indeed feel better represented by disabled candidates. This representational link does not require a sense of group identity and is only partly explained by perceptions of shared policy preferences. The study also reveals that non-disabled people feel better represented by non-disabled candidates. The findings highlight the relevance of disability as a political identity, bolstering calls for more disabled people in politics, and might help explain the disability gaps in political trust and participation.

**Supplementary Information:**

The online version contains supplementary material available at 10.1007/s11109-024-09969-z.

## Introduction

Disabled people^1^ are amongst the most politically under-represented groups in society. While around 1 in 5 people are disabled, a population that is ever increasing not least because of its positive association with aging (Iezzoni, 2014), they rarely account for more than 1 or 2 per cent of parliamentarians according to the few estimates that exist (Waltz & Schippers, 2021; Evans & Reher, 2022). “Nothing About Us Without Us” is the clarion call of the disability rights movement, which continues to demand that disabled people be included in arenas where political decisions are made – not least with regard to issues that directly affect their lives (Charlton, 1998). This slogan expresses the belief that disabled people are uniquely placed to represent the interests and concerns of the group – a group which remains marginalized in many ways and whose economic opportunities, social integration, and access to all areas of public and private life lag behind those of non-disabled people (Prince, 2009; Oliver, 2013). On this basis, we might assume that disabled people prefer to be represented by disabled politicians. At the same time, the group is heterogeneous and dispersed, and widespread stigma can make self-identifying as disabled difficult. As such, the answer to the question whether disabled citizens feel that disabled candidates represent people like them is anything but obvious.

Previous research on other social groups, most importantly women and racial and ethnic groups, has found evidence for in-group favoritism in representation perceptions as well as the vote choice (e.g. Barreto, 2007; Dolan, 2008; Goodyear-Grant & Tolley, 2019). Yet, scholars offer different explanations for these effects: some argue that, in the absence of detailed information about candidates’ policy preferences, voters use identity as a heuristic and assume that candidates who ‘look like them’ also share their preferences (e.g. Paolino, 1995; McDermott, 1998). Others insist that mere membership of a social group is not sufficient but that a positive identification with the group is necessary for a representational link to exist (e.g. Huddy, 2013; Bejarano et al., 2021). The existence and nature of a unified group identity among disabled people remains contested (e.g. Jenks, 2019; Watson, 2002), and previous research suggests a high degree of variation across individuals driven by both individual factors, such as the nature of one’s impairment, as well as contextual factors, such as societal stigma (e.g. Nario-Redmond et al., 2013; Thorp n.d.). Therefore, in addition to investigating whether disabled citizens feel better represented by disabled candidates, we also test whether this attitude is based on perceptions of shared political preferences and whether it requires a sense of group identity.

We use data from a survey experiment which presented around 6,000 respondents from the United Kingdom and the United States with descriptions of fictitious candidates who vary in terms of disability. The results show that both disabled citizens who do and those who do not identify with the disability community feel better represented by disabled candidates – albeit the former more strongly. Among citizens with a disability identity, this is partly because they believe that disabled candidates are more likely to share their policy priorities and ideological views. Importantly, however, expectations about substantive representation do not fully explain their representation perceptions, and among those without a disabled group identity this mechanism does not apply at all. Therefore, other factors, such as affective orientations, may drive these perceptions. This may also apply to non-disabled citizens, who feel better represented by non-disabled candidates, but not because they expect them to share their policy preferences.

The study highlights the political relevance of a social characteristic which has thus far been largely sidelined by political scientists as well as politicians, parties, and pundits ﻿(Evans, 2022; Erkulwater, 2006). It demonstrates the importance of enhancing diversity in, and access to, politics in order to make disabled citizens feel better represented. This might also help tackle the disability voting and trust gaps which scholars have observed in various contexts (e.g. Reher, 2020; Schur & Adya, 2013; Schur et al., 2002). The study also contributes to broader debates about political representation, demonstrating that citizens value descriptive representation not only because they expect it to improve the substantive representation of their policy preferences.

## Shared Identity, Representation Perceptions and Candidate Support

Applying Pitkin’s (1967) famous concepts of *descriptive* and *substantive representation* to the context of marginalized groups, Phillips (1995) and Mansbridge (1999) powerfully argued that members of a social group are, under certain circumstances, better at representing the interests of the group. Subsequently, a large literature has empirically tested whether the presence of members of a social group among legislators and decision-makers can advance the substantive representation of group interests and preferences in policy and outcomes. Initially it focused mostly on gender (e.g. Schwindt-Bayer & Mishler, 2005; Wängnerud, 2000) and race and ethnicity (e.g. Griffin & Newman, 2007), but has increasingly branched out to study other groups, such as education strata (e.g. Schakel & Hakhverdian, 2018), the LGBT + community (Reynolds, 2013), and also disabled people (Reher, 2022). In many cases, political representatives of a particular social group are indeed more likely to share the preferences of citizens of the group, make claims on their behalf, and increase the representation of the group’s interests in policy outcomes.

However, to what extent citizens *believe* that representatives who share their social identities are better at representing them is a separate question. Citizens may care about being descriptively represented for other reasons besides substantive representation, including conveying the group’s ability to rule (Mansbridge, 1999). The crucial question is then *which* representatives citizens perceive as representing people ‘like them’. As Celis and Childs (2020: 63–64) argue:

who counts as a descriptive representative is not read off from ‘known’ or ‘objectively observed’ ascriptive identities but remains an empirical question. … If we want to know whether she is descriptively represented, we can ask whether she sees representatives ‘like herself’ when she looks at the representative sitting on the parliamentary benches.

To develop a theoretical framework and hypotheses about the links between shared disability and representation perceptions, we can draw on research on other social groups. The literature on affinity voting provides evidence that voters indeed tend to prefer candidates who share their identity, for example their gender (e.g. Plutzer & Zipp, 1996; Dolan, 1998, 2008; Herrnson et al., 2003) or race and ethnicity (e.g. Barreto, 2007, 2010; Goodyear-Grant & Tolley, 2019; Fisher et al. 2015; McDermott, 1998; McConnaughy et al., 2010). There is, however, some variation in the explanations proposed for these patterns. While they do not fall into entirely separate theoretical camps, we can identify two distinct approaches or mechanisms: one rests on voters’ assumptions that candidates who share their attributes also share their political preferences, while the other sees an identification with the social group as necessary for voters to feel better represent by in-group candidates.^2^ Below we discuss these approaches in more detail and explore to what extent they are likely to apply to the case of disability.

### Preference Stereotypes

The first approach assumes that voters want representatives who share their political preferences and are likely to advocate for the policies they desire. Since most voters have limited time, cognitive capacity, and willingness to gather and process information about candidates’ policy positions and priorities, they often use cognitive heuristics, or information short-cuts (Lau & Redlawsk, 2001; Popkin, 1991). Drawing primarily on evidence from survey experiments, a large literature shows that voters rely on stereotypes about candidate characteristics as cues about their political views, especially in low-information contexts. For example, voters tend to perceive minority ethnic politicians as more liberal (e.g. Sigelman et al., 1995; McDermott, 1998) and women politicians as more concerned about and competent on ‘feminine issues’ (e.g. Huddy & Terkildsen, 1993; Schneider & Bos, 2014). On this basis, some scholars argue that voters tend to favor candidates who share specific attributes with them because they assume that these candidates are more likely to advocate for their political preferences (Herrnson et al., 2003; Paolino, 1995; McDermott, 1998). Importantly, for this mechanism to apply citizens do not necessarily need to strongly identify with the social group in question, as the attribute – in this case disability – functions primarily as a cognitive cue for judging whether the candidate is likely to hold similar preferences.

Based on previous evidence on the preferences of disabled citizens and elites as well as stereotypes about disabled candidates, we expect that this mechanism may indeed be at work here, leading disabled voters to feel better represented by disabled candidates. Several studies have shown that being disabled affects citizens’ policy preferences and priorities: disabled citizens tend to have more egalitarian views and higher support for state intervention in the economy, public spending, and income redistribution (Gastil, 2000; Schur & Adya, 2013; Reher, 2022; see also Mattila et al., 2017: 107ff on health). These views are likely to be shaped by their experiences of barriers in education and the labor market, lower levels of employment and income, and reliance on disability support payments, which can make it “difficult to be a model of rugged individualism and economic success” (Gastil, 2000: 591). Disabled people also tend to show stronger support for public healthcare (Gastil, 2000; Reher, 2022; Schur & Adya, 2013), state support for education and housing, and civil liberties (Schur & Adya, 2013). Some (Mattila et al., 2017; Reher, 2022), though not all (Schur & Adya, 2013), studies also found disabled people to be more left-wing.

Evidence from the UK suggests that disability preference gaps very similar to those amongst citizens exist among politicians, too: they tend to be more in favor of healthcare spending and public spending in general (Reher, 2022). Even more importantly for citizens’ representation perceptions, voters *perceive* disabled candidates to be more left-wing and more concerned about healthcare, minority rights, and welfare policy than non-disabled candidates – at least in contexts where little information about the policy agendas and positions of election candidates is available (Evans & Reher, 2024). Based on these observations, we would expect disabled voters to perceive disabled candidates as more likely to represent them than non-disabled candidates. Similarly, we would expect non-disabled voters to feel better represented by non-disabled candidates, since they perceive disabled candidates’ preferences to be more distant. If the preference stereotype-based mechanism is indeed at work, we would expect these representation perceptions to be largely explained by perceptions of shared policy preferences.

### Group Identity

Proponents of the second approach do not necessarily reject the idea that beliefs about shared preferences can be an important factor in voters’ preference for in-group candidates, but they insist that an identification with the social group is at the root of it and a necessary condition for in-group favoritism in candidate preferences and voting (e.g. Bejarano et al., 2021; Gershon et al., 2019; Plutzer & Zipp, 1996; Dolan, 2008). Looking at political cohesion more generally, Huddy (2013: 744) explains that:

Membership in a social group does not necessarily prescribe a specific political outlook, nor does it dictate political action on a group’s behalf. Several factors are central to the development of political cohesion: the existence of strong identities, convergent identities, the political meaning of group membership, the existence of symbolic and realistic threats and grievances, and group consciousness.

In the same vein, Bejarano et al. (2021) argue that sharing an identity or demographic alone does not explain a preference for a candidate; it necessitates group identification and consciousness. Goodyear-Grant and Tolley (2019), for example, find that only Chinese Canadians whose self-identity is sensitive to their group being criticized demonstrate a pattern of same-race voting, while mere self-categorization into the group is not sufficient.

Group identification is argued to drive preferences for in-group political representatives through various mechanisms. The concept of linked fate, which originates in scholarship on group consciousness among African Americans (Dawson, 1994; Tate, 1994), refers to the belief that what happens to the group affects each individual in the group. Scholars have used it to explain preferences for in-group candidates among Black and Latino citizens in the US (e.g. Casellas & Wallace, 2015; Tate, 1994; Barreto, 2010; McConnaughy et al., 2010) as well as intersectional (Bejarano et al., 2021; Gershon et al., 2019) and panethnic linked fate (Sanchez & Masuoka, 2010). It posits that group identification drives perceptions of shared political interests; however, citizens’ desire to be represented by in-group members may also be driven by “cultural forces or extra-policy goals” (Gay, 2002: 718). Members of minoritized groups may connect emotionally to in-group politicians (Dolan, 2008), place greater trust in them (Tate, 2003; Casellas & Wallace, 2015), perceive them as more accessible, receptive, and responsive (Fenno, 1978; Gay, 2002), value the positive signals they send about the group’s legitimacy in positions of power (Bobo & Gilliam, 1990; Mansbridge, 1999), and see them as countering cultural threats (Goodyear-Grant & Tolley, 2019).

### A Disabled Group Identity?

Whether a unified disability exists, and what its nature is, remains highly contested among both scholars studying disability and disability activists (Jenks, 2019). Disabled people are an extremely heterogeneous group, more so than many other social groups. There are a wide range of impairment types, including physical, sensory, and cognitive impairments, neurodivergence etc., all of them with different levels of severity. These impairments interact with people’s environments and intersecting identities, such as social class, gender, ethnicity etc., to create an even greater range of barriers. As a result, disabled people can have vastly different experiences from each other (Fine & Asch, 1988). As such, Putnam (2005: 195) argues, “disability, as a minority characteristic, may be thought of as substantively different than gender, race, ethnicity, or sexual orientation as it is more loosely defined and somewhat more malleable based on political concern”. In his comparative study of the disability rights movement, Charlton (1998: 78) argues that one of the key challenges facing the movement is the ‘failure’ of disabled people to identify as such.

At the same time, many disabled people do have common experiences, which are often manifestations and results of ableism – the expectation that individuals adjust and conform to society’s able-bodied/minded ideal (Goodley, 2016). These experiences include being marginalized or excluded from certain areas of public and private life and treated with prejudice and neglect. While the specific adjustments disabled people require, for example to perform a job or participate in an event, may be different, they share a need for accessible spaces and institutions (and legislation to ensure it). If, as a result of these shared experiences, disabled people perceive within-group differences to be less pronounced than between-group differences, the disabled community may become a salient social identity for them (Turner et al., 1987).

Social Identity Theory posits that developing a strong identification with one’s in-group can be a strategy to counter stigma and discrimination (Tajfel & Turner, 1979; Turner et al., 1987), and indeed, research suggests that having a disabled group identity is often experienced as positive and associated with higher self-esteem (Hahn & Belt, 2004; Nario-Redmond et al., 2013; Putnam, 2005). However, this is only one possible reaction to stigma: individuals who have internalized ableism and seek to avoid being ‘othered’ or seen as vulnerable, weak, and dependent may also reject their disabled identity and instead ‘normalize’ (by denying the relevance of their disability) or ‘pass’ (as non-disabled) (Anspach, 1979; Dirth & Branscombe, 2018; Nario-Redmond et al., 2013; Goffman, 1963). Thus, while some people take pride in the term ‘disabled’ and draw confidence from their group identity, others reject membership of the group, or at least the notion that it shapes their identity (Engel & Munger, 2003; Watson, 2002; Barnes et al., 1999).

In other cases, rather than explicitly rejecting the idea of belonging to the disability community, individuals may simply not have had the opportunity to develop an awareness of shared experiences and, hence, a group identity. As Dunn and Burcaw (2013: 149) point out, a “recurring theme in the formation of disability identity is the importance of community, where people with disabilities are actively engaged with their peers due to common experience.” Yet, not all disabled people have had the opportunity to be around other disabled people or become involved in disability activism and organizations. They are often the only disabled individuals in their family and immediate social context (Dunn & Andrews, 2015; Scotch, 1988). Several factors may facilitate network building, for example attending special schools, living in long-term care facilities (Scotch, 1988), or having access to social media, which is nowadays a common way for disabled people to get into contact with each other, learn about the community, and become mobilized into disability advocacy (Webster, 2022). The wider context may play a role, too, including the existence and prominence of a disability rights movement in a country, variation in bureaucratic approaches to the definition, which can emphasize certain types of disabilities, and of course the stigma surrounding disability and being disabled (Iezzoni, 2000; Schur et al., 2013).

The intersections with individuals’ other social identities, such as gender identity, race, social class etc., are also likely to affect individuals’ disability identity (Erevelles, 2011; Schalk, 2022). All of us belong to various social categories, and different ones may become salient at different times, due to a variety of reasons (Young., 1990). For example, individuals’ disability identity might be particularly salient, and politically consequential, when it is more strongly politicized or when they perceive the group to be under threat, as we mentioned above and discuss further in the conclusion. In addition, some disabled people might feel marginalized within the disability community, for example due to their race, and struggle to identify with it as a result.

There are a few existing studies on disability identity from the US, but they mostly draw on small and/or unrepresentative samples which do not allow us to draw inferences to the wider (disabled) population. Still, this research finds that while many disabled people do indeed identify as part of a wider disability community, others do not. They suggest that the nature of the impairment can affect this, including its duration, origin, visibility, severity, and number of impairments (Bogart, 2014; Bogart et al., 2017; Nario-Redmond & Oleson, 2016; Hahn & Belt, 2004; Nario-Redmond et al., 2013; Olney & Brockelman, 2003; Thorp n.d.). Experience with workplace or school accommodations and government assistance as well as experiences of discrimination also strengthen disability group identity (Thorp n.d.). In sum, it appears that disability does constitute a relevant social identity, but there is considerable variation in the extent to which individuals who identify as disabled perceive it as a group identity.

There is even less existing research into the political implications of holding a disability identity, but a few findings are worth pointing out here. Thorp (n.d.) finds that disabled people with a strong disability group identity are more likely to have a sense of linked fate and to believe that disabled people have shared political interests than those with a weak identity, which supports the argument that social identity is key for group-based feelings of political representation. Meanwhile, Nario-Redmond and Oleson (2016) suggest that identifying with the disability community is strongly correlated to participation in organizations with other disabled people and involvement in groups advocating disability rights.

### Hypotheses

On the basis of the preference stereotypes and identity approaches, we formulate a set of hypotheses about how candidate and citizen disability interact in shaping citizens’ perceptions of how well candidates represent them. According to the idea that candidate disability serves as an information shortcut for candidates’ policy preferences we would expect that:

#### Hypothesis 1

*Disabled citizens feel better represented by disabled than non-disabled candidates*,* while non-disabled people feel better represented by non-disabled candidates.*

**Fig. 1:**
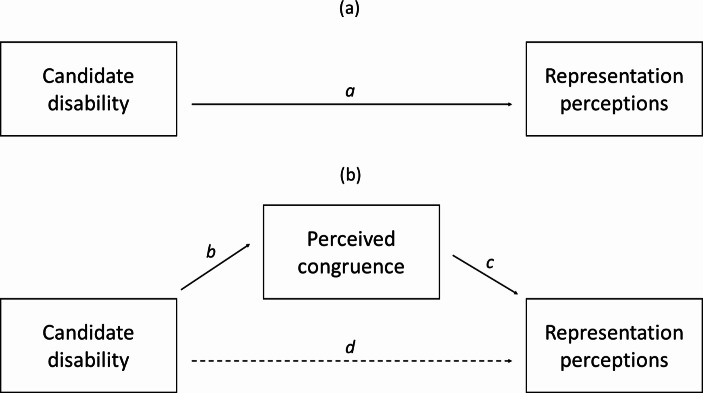
Hypothesized relationships between candidate disability and representation perceptions, with mediating effect of perceived congruence

In other words, effect *a* in the top pane (a) in Fig. 1 should be positive among disabled and negative among non-disabled citizens. The preference stereotypes approach suggests moreover that perceptions of shared preferences with candidates are the key mediating factor linking candidate disability with representation perceptions:

#### Hypothesis 2

*Perceptions of shared policy preferences mediate the effect of candidate disability on representation perceptions.*

This is illustrated by paths *b* and *c* in part (b) of Fig. 1, which explain most or all of the effect of candidate disability on representation perceptions (path *d*).

The identity-based approach suggests additional and to some degree conflicting expectations. First, based on the argument that mere self-categorization as disabled without a group identity is not sufficient to generate a sense of shared political interests, we would expect the following:

#### Hypothesis 3

*Only disabled citizens who identify with the disability community feel better represented by disabled candidates.*

Moreover, the approach argues that while group identification can lead to a sense of shared interests, there are also other, non-policy related mechanisms that link identity to a preference for representatives with a shared identity:

#### Hypothesis 4

*Among disabled group identifiers*,* not the entire effect of candidate disability on representation perceptions is mediated by congruence perceptions.*

In other words, a positive effect of candidate disability on representation perceptions (path *d*) is likely to remain among disability group identifiers when we account for paths *b* and *c*.

## Data and Methods

We test the hypotheses through a survey experiment embedded in an online survey of representative samples (based on age, gender, and regional quotas) of around 3,000 respondents in the United Kingdom and around 3,000 respondents in the United States, fielded by Qualtrics in May-June 2020 and January 2021 (Reher, 2024). To be sure, there are important differences between the two cases, for example the US operates a two-party and the UK a multi-party system, although the latter is dominated by two major parties. The UK’s National Health Service, which is free at the point of delivery, differs from the for-profit system in operation across the US. And yet, despite these differences the UK and the US are very suitable cases for the study as they have candidate-centered, majoritarian electoral systems, which means that citizens are used to evaluating individual candidates. Voters in the two countries are likely to be familiar with the idea of disabled candidates, as both have had several prominent disabled politicians on both the left and the right (including former MPs Anne Begg, David Bluckett, and Robert Halfon and current MP Marsha de Cordova in the UK, and current Senator Tammy Duckworth and Governor of Texas Greg Abbott as well as former Congressman James Langevin and former President Franklin D. Roosevelt in the US). As a result, the risk of demand effects, where respondents guess the purpose of the experiment and respond in a way that confirms the hypotheses, might be lower than in countries where citizens have not witnessed disabled politicians before.

Respondents were presented with two sets of short vignettes describing two fictional candidates, A and B, competing for election to the House of Commons (UK) or House of Representatives (US) in their constituency or district. The vignettes either do not mention disability or state that the candidate is (i) paralyzed below the waist and uses a wheelchair to get around, (ii) blind and reads using text-to-speech software, or (iii) D/deaf and communicates mostly in British/American Sign Language. By specifying how the candidates address the respective barriers they face, respondents are provided with information about the nature of the main barriers – mobility, reading, and communicating, respectively – and the adjustments they use, which provides them with cues about what the disability might mean for their election campaigns and their work as representatives.

The vignettes also contain information about a number of other attributes of the candidates, whose values are randomly assigned in a conjoint design: gender, minority ethnic status, age, profession, number of children, years of political activity, and previous experience of elected office at a lower level of government.^3^ Including this wider set of attributes allows us to estimate the effects of candidate disability for a range of different hypothetical candidates. Moreover, including a range of characteristics is likely to further reduce demand effects, as candidate disability is only one attribute amongst several which the researchers might be interested in. Finally, it helps reduce the risk that respondents provide answers they consider socially desirable, as their evaluations of the candidates could be driven by a number of factors besides disability. Each respondent saw one set of vignettes which did not mention political parties and another set where the two candidates stood for the Labour and Conservative Party (UK) or the Democratic and Republican Party (US). The order in which respondents saw these two sets of vignettes was randomized. Each respondent thus evaluated four candidates in total.

After being presented with a pair of vignettes, respondents were asked a set of questions about their impressions of both candidates, including “How much do you agree or disagree with the following statements about the two candidates? Candidate A[B] represents people like me” and responded on a scale from 1 = strongly disagree to 5 = strongly agree. This measure of representation perceptions was normalized to range from 0 to 1 and is the main dependent variable in the analysis.

Respondents were also asked a set of questions about their own disability status and group identity, starting with: “Do you have any long-term illness, mental health problem or disability which limits your daily activities or the work you can do?” Those who indicated being disabled were then also asked “Do you think of yourself as belonging to the disability community?”, which constitutes the group identity measure.^4^ We asked these questions at the end of the survey, after the experiment, to avoid priming respondents on their disability and thereby making this identity more salient when they evaluate the candidates. This should also reduce the risk of demand effects and social desirability bias. However, the trade-off is the risk of potential post-treatment bias, which can occur when conditioning variables are measured after the treatment is assigned and are themselves affected by the treatment (Montgomery et al., 2018). In this case we anticipated that the risk that respondents’ answers about their own disability status and identity are affected by the experimental treatments would be lower than the risks of priming and social desirability bias. Still, it is in principle possible that, for example, respondents who saw one or several disabled candidates felt more comfortable declaring their own disability. The SI 5 discusses this risk and the potential implications in detail.

**Table 1:** Frequencies of disability, disability types, and disability identity in the samples

	UK			US		
	Number of respondents	Percentage among all respondents_1_	Percentage among disabled respondents_1_	Number of respondents	Percentage among all respondents_1_	Percentage among disabled respondents_1_
Disabled	738	25.3		862	29.3	
Disabled identity	189	6.7	28.7	298	10.5	39.3

_1_ Calculations were made excluding respondents who preferred not to answer the disability question (78 respondents in the UK, 75 in the US) or the disability identity question

Table 1 shows that 25.3 per cent of British and 29.3 per cent of US survey respondents indicated that they are disabled. These are only slightly higher than official figures: an estimated 22 per cent of the UK population were disabled in 2020/21 (Kirk-Wade, 2022), while the estimate was 24.8 per cent in the US in 2020 (Centers for Disease Control and Prevention 2022). With 738 respondents in the UK and 862 respondents in the US indicating that they are disabled, we have a substantial group size. Among the respondents who said that they are disabled, 28.7 per cent in the UK and 39.3 per cent in the US indicated that they identify with the disability community. This is an interesting difference and further research could usefully explore and explain this disparity.

To test the mediating effect of perceptions of shared policy preferences we construct a measure of perceived preference congruence between individual respondents and candidates. Before the experiment, respondents were asked about the importance they place on six policy issues: social security/welfare; military and defense; healthcare; minority rights; economy; and family and children. They were also asked about their position on the left-right dimension. After seeing the vignettes, respondents were asked how important they thought the same issues were to the candidates, and to place them on the left-right dimension. We subtracted respondents’ perception of each candidate’s placement from their own on each dimension, and the absolute value of each difference was subtracted from 1 to measure perceived congruence.^5^ The result is a set of 7 congruence perception measures where 0 = lowest congruence and 1 = highest congruence. The priority congruence and left-right congruence measures were then averaged into a *perceived congruence index*.

## Results

To test Hypotheses 1 we estimate the Average Marginal Component Effect (AMCE) (Hainmueller et al., 2014) of candidate disability on citizens’ feeling of whether a candidate represents people like them. We summarize the three impairment types into one disability category and pool the data from the UK and the US, but the SI 2 includes analyses by country and disability type^6^. The analyses regress representation perceptions on all candidate attributes that were randomized and interact the binary measure of candidate disability with a three-category disability identity measure: (1) non-disabled, (2) disabled and no disability group identity, and (3) disabled and disability group identity.

**Fig. 2:**
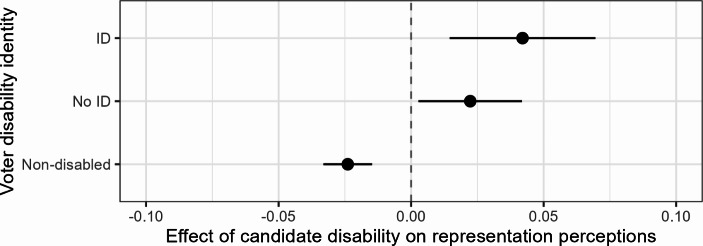
Effects of candidate disability on representation perceptions, by disability identity (ID). *Notes*: Full estimates in Table S2 (Model 2)

Figure 2 shows the conditional effects of candidate disability, where positive coefficients mean that respondents feel better represented by disabled candidates and negative coefficients mean that they feel better represented by non-disabled candidates. We see that disabled citizens both with and without a disability group identity (ID) perceive disabled candidates as more representative of themselves than non-disabled candidates, supporting Hypothesis 1. The effect is stronger among disabled citizens with a disability identity (0.42 points on the 0–1 scale) than those without (0.22), but the two effects are not statistically significantly different from each other. This means that Hypothesis 3 – the expectation that only those with a disability identity have an in-group affinity in terms of representation – is not supported. Meanwhile, non-disabled citizens perceive disabled candidates to be less representative of people like them than non-disabled candidates, and this effect (-0.24) is statistically significantly different from the effects among both groups of disabled citizens.

**Fig. 3:**
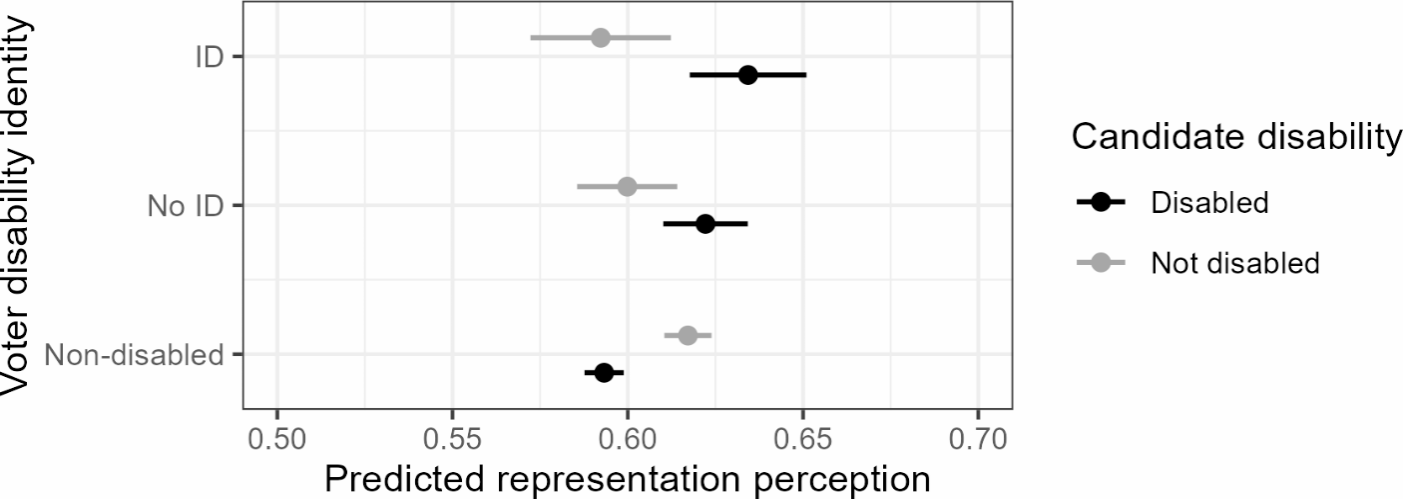
Predicted representation perceptions of disabled and non-disabled candidates, by disability identity. *Notes*: Based on Model 2 in Table S2 and Fig. 2. Covariates held at their sample means and proportions

Figure 3 shows the fitted values on the representation perception scale based on these estimates. It demonstrates that especially citizens who identify with the disability community feel better represented by disabled candidates than non-disabled citizens feel represented by non-disabled candidates. Non-disabled citizens feel similarly well represented by disabled candidates as disabled citizens feel represented by non-disabled candidates, just below 0.6 on the 0–1 scale.

To give a sense of the magnitude of the effects of candidate disability in comparison to other identities, we estimate a model interacting candidate gender with citizen gender. The effect of candidate gender differs statistically significantly between women and men: among women, the effect of candidates being described as female rather than male on their representation perceptions is 0.016 (*p* < 0.01). This is smaller than the effects of candidate disability among disabled citizens with and without a group identity. Meanwhile, candidate gender does not affect men’s representation perceptions. This comparison to a well-known factor in candidate evaluations underlines the relevance of disability as an identity in the political representation process.

We proceed by examining to what extent these patterns of in-group affinity in representation can be explained by perceptions of shared policy preferences. As explained above, we construct the preference congruence measure by calculating the average proximity of each individual respondent to each of the candidate profiles they saw with regard to six issue priorities and the left-right dimension.^7^ To examine the potential mediating effects of preference congruence we conduct mediation analyses using the framework by Imai et al. (2010) and the mediation package in R (Tingley et al., 2014). This involves estimating (i) the mediator model, which estimates the effect of the treatment (candidate disability) on the mediator (perceived congruence); (ii) the outcome model, which regresses the outcome (representation perceptions) on both the mediator and the treatment; and (iii) the Average Causal Mediation Effect (ACME), the Average Direct Effect (ADE), and the Total Effect of the treatment on the outcome. We analyze non-disabled citizens, disabled citizens without a disabled group identity, and disabled citizens with a group identity separately. The full results are shown in the SI 4 (Tables S5-S8).

**Fig. 4:**
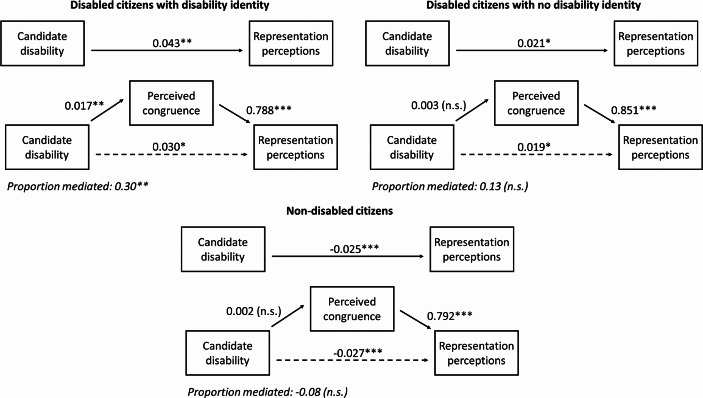
Mediation analyses of perceived congruence mediating the effect of candidate disability on representation perceptions. *Notes*: The full model estimates are shown in Tables S5, S6, and S7. The proportions mediated are shown in Table S8. *p < 0.05, **p < 0.01, ***p < 0.001, n.s.=not statistically significant

Figure 4 illustrates for each group of citizens the total effect of candidate disability on representation perceptions^8^, *a*, as well as the effect of candidate disability on perceived congruence *b* from the mediator model, the effect of perceived congruence on representation perceptions *c* from the outcome model, and the remaining direct effect of candidate disability on representation perceptions *d* from the outcome model. Looking first at disabled citizens with a disability identity, around 30 per cent of the total effect of candidate disability on representation perceptions is mediated by perceptions of shared preferences. Disabled citizens who identify with the disability community perceive disabled candidates to be around 0.02 points on the 0–1 congruence scale closer to their own political views than non-disabled candidates, and this makes them more likely to see them as representatives of people like them. While this supports Hypothesis 2, we also find support for Hypothesis 4: even when holding perceived preference congruence constant, this group of disabled citizens still feel better represented by disabled candidates. This suggests that affective orientations, such as trust, solidarity, and in-group favoritism, are also at work here.

Among disabled citizens who do not feel part of a wider disability community, the overall positive effect of candidate disability on feelings of being represented is about half as large, at 0.021 on the 0–1 scale, although recall from Fig. 2 that the effects did not differ statistically significantly between the group identifiers and non-identifiers. Contrary to Hypothesis 2, this effect is not mediated by preference congruence: disabled citizens who do not identify with the disability community do not believe that disabled candidates are more likely to share their policy preferences. To some extent this finding echoes Thorp’s (n.d.) observations that disabled people with a weak disability group identity are less likely to have a sense of linked fate and to believe that disabled people have shared political interests than those with a strong identity. The finding that disabled citizens without a group identity still feel better represented by disabled candidates is, therefore, likely due to ‘extra-policy’ factors such as greater trust – something we had only expected from those with a group identity.

Finally, we test whether the finding that non-disabled citizens feel better represented by non-disabled candidates – 0.025 points on the 0–1 scale – can be explained by perceived preference congruence. Interestingly, this is not the case: non-disabled citizens do not believe that non-disabled candidates better reflect their own policy priorities and ideology, but they still feel better represented by them. Again, this finding contradicts the preference stereotype approach, instead suggesting that affective orientations towards non-disabled and disabled candidates might be at play here as well.

## Conclusion

Identity politics has become a much-debated concept in recent years, with some complaining that social groups and divisions are taking precedent over political beliefs and ideas, and others welcoming the recognition that shared experiences can be a foundation for fighting injustices. Disability is not an identity that has been at the center of these debates, and we rarely see the claims and demands of the disability rights movement expressed in electoral campaigns and parliaments. Still, disabled people constitute a key minority group in societies around the world – one that has historically suffered oppression and marginalization and is still fighting for full rights and equality. Moreover, the disability population is rapidly increasing: according to the World Health Organization (n.d.) this is partly due to aging but also to the rise in noncommunicable diseases, thus underscoring the urgent need for political actors to adequately represent their interests. This raises the question of whether disabled citizens feel better represented by disabled politicians. In answering this question, our study builds on previous research on voter perceptions of disabled candidates (Evans & Reher, 2024) by shining a light on how citizens’ own disability status and identity shape such perceptions.

Our findings show that disabled citizens indeed feel better represented by disabled candidates. To what extent are these perceptions driven by a group identity, and to what extent by an expectation that disabled candidates are more likely to share and promote their substantive policy preferences? Among those who identify with the disability community, both mechanisms seem to be at work: they feel better represented by disabled candidates in a *substantive* sense, but also in a way that is unrelated to policy preferences. These perceptions are presumably driven by a sense of solidarity, trust, and a desire for visibility of disabled people in positions of power – in Pitkin’s (1967) words, they may feel *symbolically* represented by disabled candidates.

Against our expectations, disabled citizens who lack an identification with the disability community also feel better represented by disabled candidates, and this is not explained by a sense of shared policy preferences. Thus, it seems that a strong group identity might not be necessary for citizens to prefer politicians who share their characteristics. In the case of disability, one explanation might be that many disabled people who do not say that they feel part of a disability community do not explicitly reject the identity, but rather have not had opportunities to develop relationships with other disabled people. Many of them might in fact like to belong to such a community of shared experiences and goals, and for this reason feel represented by disabled candidates.

A final important observation is that non-disabled citizens feel better represented by non-disabled than disabled candidates, and this sentiment is not explained by perceived preference congruence. This suggests that disability is perceived as a politically relevant identity not only by disabled people but also by non-disabled people. It might mean that non-disabled citizens do not trust disabled candidates to care about their interests and needs, even if they do not expect them to focus on different policy areas or hold different ideological views. However, it might also mean that many non-disabled people perceive disabled people as a socially distant out-group. The finding is particularly noteworthy given that the disability types we studied – physical and sensory disabilities – are not as strongly stigmatized as, for example, cognitive disabilities or mental health conditions. At the same time, they are well-known disabilities and likely to be seen as permanent. As such, they might be more readily interpreted as markers of identity than, for example, chronic illnesses or mental health conditions. Future research should explore both disabled and non-disabled group identity in more detail, as well as examining a wider range of disability types.

It is important to note that representation perceptions do not necessarily directly translate into candidate support. Indeed, there is evidence that citizens see disabled candidates as bringing a range of traits and skills to the table, such as being more compassionate, honest, and hard-working, and being more competent on issues including healthcare, minority rights, and welfare (Evans & Reher, 2024). Some voters might also appreciate candidates who do not represent people like themselves but marginalized and under-represented groups. Thus, it would be premature to conclude from these findings that non-disabled citizens are unwilling to vote for disabled candidates.

The intersections with other identities also require further research. For example, would disabled citizens struggling to get by on benefits feel represented by a disabled candidate with a high-earning job, or would they feel better represented by a non-disabled politician with lived experience of the welfare system? And how do the intersections with other politicized identities such as race affect the representational dynamics? Our survey was conducted during the Covid-19 pandemic, when public health dominated the debate and many disabled people felt threatened both by the virus itself and policies regarding distancing and issues such as ‘do-not-resuscitate orders’ (Trapper, 2021). Thus, disability identity might have been particularly salient at the time. Future research should examine how the political context, for example the extent to which a group is under threat by policies or other societal forces (cf. Huddy, 2013), as well as politicians’ own representative claims can shape which identities citizens seek out in their representatives. The role of political parties needs to be considered here as well; given that disabled candidates are stereotyped as left-wing, disability identity might be considered less salient among right-wing politicians.

## Electronic Supplementary Material

Below is the link to the electronic supplementary material.

Supplementary Material 1

## Data Availability

Replication files can be accessed in the Journal’s Dataverse at 10.7910/DVN/QP50GA.

## Footnotes

1 We recognize that there is a debate over language when it comes to disability. Some prefer person-first terminology (people with disabilities) whereas others choose identify-first language (disabled people); these choices vary across, as well as within, countries. There are good arguments to support the choice of either linguistic approach. We have chosen to use identity-first language to emphasize the barriers that exist in society which disable an individual, rather than disability being inherent to the person. Ultimately, we agree with Andrews et al. (2022) that the most important aspect is that it does not stigmatize disabled people.

2 Similar distinctions have been formulated by Goodyear-Grant and Tolley (2019) and Casellas and Wallace (2015).

3 The values of all attributes are shown in Tables S1 and S2 and the full vignettes in Figure S1 in the Supplementary Information.

4 While the item mostly taps into the cognitive dimension of identity since it asks respondents to ‘think’, the aspects of ‘belonging’ and ‘community’ do hold affective and positive evaluative connotations (cf. Tajfel, 1978). As Huddy (2013) explains, a sense of belonging to a group is an essential marker of group identification and social identity. It goes beyond mere group membership based on objective criteria, which our disability status item measures.

5 Respondents’ issue importance was measured on 11-point scales, whereas their perceptions of candidates’ issue importance was measured on 5-point scales. Ideology was measured on 11-point scales, from 0 = left to 10 = rights. All scales were normalized to range from 0 to 1.

6 The estimates are very similar in the two countries. The analyses by disability type suggest that the feelings of representation among disabled citizens are stronger for the wheelchair-using candidate. The SI 2 discusses this in more detail.

7 Sect. 3 of the SI includes detailed analyses of the two components of the measure: citizens’ perceived policy preferences of disabled and non-disabled candidates, and differences in policy preferences between disabled and non-disabled citizens.

8 The coefficients are from the samples that exclude observations with missing values on the congruence variable and may therefore differ slightly from the coefficients in Fig. 2.
