# Supplementary material for: Someone Like Me? Disability Identity and Representation Perceptions
Source: Polit Behav. 2024 Aug 28;47(2):689–709. doi: 10.1007/s11109-024-09969-z (PMC12053203; doi:10.1007/s11109-024-09969-z)
Supplement: Supplementary file 1 — Supplementary Material 1 [file 11109_2024_9969_MOESM1_ESM.docx]

**Someone like me? Disability identity and representation perceptions**

***Political Behavior***

Stefanie Reher, University of Strathclyde, [stefanie.reher@strath.ac.uk](mailto:stefanie.reher@strath.ac.uk)

Elizabeth Evans, University of Southampton, [E.J.Evans@soton.ac.uk](mailto:E.J.Evans@soton.ac.uk)

**SUPPLEMENTARY INFORMATION**

**Contents**

**Page**

1. Survey experiment design 2
2. Effects of candidate disability on representation perceptions, conditional on citizen disability and group identity 4
3. Disability effects on perceived candidate preferences and citizen preferences 8
4. Mediation analysis 12
5. Analysis of relationships between candidates’ and respondents’ disability status and identity 17
6. **Survey experiment design**

| **Introduction**  Ahead of elections, voters often have only limited information about the candidates who are competing. We are interested in how people evaluate candidates in such settings.  On the next page, you will see descriptions of two fictional candidates, Candidate A and Candidate B. Imagine that a general election was coming up and that the two candidates were competing in your [constituency/district] for a seat in the [House of Commons/ U.S. House of Representatives].  We will asked a few questions about each pair of candidates. We are interested in your impressions of the candidates based on the short descriptions. Don't worry if you are unsure - there are no right or wrong answers. Remember that the survey is completely anonymous.  [next page]  **Comparison 1** [NO PARTY AFFILIATION]  Please read the descriptions of these two candidates carefully.  **Candidate A**  <NAME> is <AGE> years old and has <CHILDREN>. <PRONOUN> <JOB>. <PRONOUN><DISABILITY>. <NAME> has been politically active in your [constituency/district] for <EXPERIENCE> years. <PRONOUN> <OFFICE>.  **Candidate B**  <NAME> is <AGE> years old and has <CHILDREN>. <PRONOUN> <JOB>. <PRONOUN><DISABILITY>. <NAME> grew up in your [constituency/district] and has been involved in politics for <EXPERIENCE> years. <PRONOUN> <OFFICE>.  **Comparison 2** [PARTY AFFILIATION]  Please read the descriptions of these two candidates carefully.  **Candidate A**  <NAME> is the <PARTY> Party candidate in your [constituency/district]. <NAME> is <AGE> years old and has <CHILDREN>. <PRONOUN> <JOB>. <PRONOUN><DISABILITY>. <NAME> has been politically active in your [constituency/district] for <EXPERIENCE> years. <PRONOUN> <OFFICE>.  **Candidate B**  <NAME> is the <PARTY> Party candidate in your [constituency/district]. <NAME> is <AGE> years old and has <CHILDREN>. <PRONOUN> <JOB>. <PRONOUN><DISABILITY>. <NAME> grew up in your [constituency/district] and has been involved in politics for <EXPERIENCE> years. <PRONOUN> <OFFICE>. |
| --- |

**Figure** S**1.** Introduction and vignettes (no party affiliation example)

*Notes:* The order of ‘party affiliation’ and ‘no party affiliation’ experiments (i.e., appearing in Comparison 1 or Comparison 2) is randomized.

**Table S1.** Attributes and values

| **Attribute** | **Values [probability or restriction]** |  |
| --- | --- | --- |
|  | **US** | **UK** |
| Name:  Gender,  Immigration background | *Pair 1: Candidate A:* “Paul Smith” [0.35], “Anna Smith” [0.35], "Sofia García" [0.15], "Carlos García” [0.15]  *Pair 1: Candidate B:* "Ian Wright" [0.35], "Jane Wright" [0.35], "Valeria López" [0.15], "Jorge López " [0.15]  *Pair 2: Candidate A:* "David Jones" [0.35], "Mary Jones" [0.35], "Maria Sanchez" [0.15], “Marcos Sanchez" [0.15]  *Pair 2: Candidate B:* "Tom Williams" [0.35], "Kate Williams" [0.35], "Gloria Ramos" [0.15], "Sergio Ramos" [0.15] | *Pair 1: Candidate A:* “Paul Smith” [0.35], “Anna Smith” [0.35], "Amita Chowdhury" [0.15], "Rahul Chowdhury” [0.15]  *Pair 1: Candidate B:* "Ian Wright" [0.35], "Jane Wright" [0.35], "Nadia Abadi" [0.15], "Samir Abadi" [0.15]  *Pair 2: Candidate A:* "David Jones" [0.35], "Mary Jones" [0.35], "Meena Jarwar" [0.15], "Dev Jarwar" [0.15]  *Pair 2: Candidate B:* "Tom Williams" [0.35], "Kate Williams" [0.35], "Leila Said" [0.15], "Masoud Said" [0.15] |
| Pronoun | “He”, “She”, *NA* [if Disability==*NA*] | “He”, “She”, *NA* [if Disability==*NA*] |
| Party | “Democratic”, “Republican” | “Labour”, “Conservative” |
| Age | 35 – 65 | 35 – 65 |
| Children | “no children", "one child", "two children", "three children" | “no children", "one child", "two children", "three children" |
| Job | "owns a small business which employs five people",  "works as an elementary school teacher",  "works in a local factory",  "works as a lawyer for a large international firm",  "works as a doctor in a local hospital" | "owns a small business which employs five people",  "works as a primary school teacher",  "works in a local factory",  "works as a lawyer for a large international firm",  "works as a doctor in a local hospital" |
| Disability | *NA* [0.4],  "is paralyzed below the waist and uses a wheelchair to get around." [0.2],  "is blind and reads using text-to-speech software." [0.2],  "is deaf and communicates mostly in American Sign Language. [0.2]" | *NA* [0.4],  "is paralysed below the waist and uses a wheelchair to get around." [0.2],  "is blind and reads using text-to-speech software." [0.2],  "is deaf and communicates mostly in British Sign Language. [0.2]" |
| Experience | 4 – 17 | 4 – 17 |
| Office | "has previously served as a state legislator",  "has not yet held elected office" | "has previously served as a local councillor",  "has not yet held elected office" |

1. **Effects of candidate disability on representation perceptions, conditional on citizen disability and group identity**

**Table S2.** Linear regression of representation perceptions on candidate disability

|  | (1) | (2) | (3) | (4) | (5) |
| --- | --- | --- | --- | --- | --- |
|  | No interaction | Fig. 2 + 3 | UK only | US only | Disability type |
| C disabled | -0.011^**^ | -0.024^***^ | -0.025^***^ | -0.023^***^ |  |
|  | (0.004) | (0.004) | (0.006) | (0.007) |  |
| *C disability* (ref=non-disabled) |  |  |  |  |  |
| C blind |  |  |  |  | -0.029^***^ |
|  |  |  |  |  | (0.006) |
| C Deaf |  |  |  |  | -0.023^***^ |
|  |  |  |  |  | (0.006) |
| C paraplegic |  |  |  |  | -0.020^**^ |
|  |  |  |  |  | (0.006) |
| *V disability identity* (ref=non-disabled) |  |  |  |  |  |
| Disabled ID |  | -0.025^*^ | -0.024 | -0.025 | -0.025^*^ |
|  |  | (0.011) | (0.019) | (0.016) | (0.012) |
| No disabled ID |  | -0.017^*^ | -0.034^**^ | 0.002 | -0.017 |
|  |  | (0.008) | (0.013) | (0.013) | (0.009) |
| C age | -0.0002 | -0.0002 | -0.0004 | 0.0001 | -0.0002 |
|  | (0.0002) | (0.0002) | (0.0003) | (0.0003) | (0.0002) |
| C female | 0.004 | 0.004 | 0.011^*^ | -0.002 | 0.004 |
|  | (0.004) | (0.004) | (0.005) | (0.006) | (0.006) |
| C minority | -0.014^***^ | -0.014^***^ | -0.028^***^ | 0.001 | -0.014^***^ |
|  | (0.004) | (0.004) | (0.006) | (0.006) | (0.004) |
| *C profession* (ref=doctor) |  |  |  |  |  |
| Factory worker | 0.005 | 0.005 | -0.004 | 0.014 | 0.004 |
|  | (0.006) | (0.006) | (0.008) | (0.009) | (0.006) |
| Lawyer | -0.049^***^ | -0.049^***^ | -0.061^***^ | -0.035^***^ | -0.049^***^ |
|  | (0.006) | (0.006) | (0.008) | (0.009) | (0.006) |
| Small business owner | -0.004 | -0.003 | -0.017^*^ | 0.011 | -0.003 |
|  | (0.006) | (0.006) | (0.008) | (0.009) | (0.006) |
| Teacher | 0.012^*^ | 0.012^*^ | 0.003 | 0.022^**^ | 0.012^*^ |
|  | (0.006) | (0.006) | (0.008) | (0.009) | (0.006) |
| C experience | 0.001^*^ | 0.001^*^ | 0.002^**^ | 0.0001 | 0.001^*^ |
|  | (0.0005) | (0.0005) | (0.001) | (0.001) | (0.0005) |
| C children | 0.012^***^ | 0.012^***^ | 0.012^***^ | 0.012^***^ | 0.012^***^ |
|  | (0.002) | (0.002) | (0.002) | (0.003) | (0.002) |
| C office | 0.009^*^ | 0.009^*^ | 0.012^*^ | 0.005 | 0.009^*^ |
|  | (0.004) | (0.004) | (0.005) | (0.006) | (0.004) |
| *C party* (ref=Democrat/Labour) |  |  |  |  |  |
| No party | -0.00004 | -0.00000 | -0.011^*^ | 0.011 | 0.00001 |
|  | (0.005) | (0.005) | (0.005) | (0.006) | (0.004) |
| Republican/Conservative | -0.038^***^ | -0.038^***^ | -0.056^***^ | -0.019^*^ | -0.038^***^ |
|  | (0.005) | (0.005) | (0.007) | (0.008) | (0.005) |
| *Interaction C disability * V disability identity* (ref=non-disabled) |  |  |  |  |  |
| C disabled * V disabled ID |  | 0.066^***^ | 0.084^***^ | 0.056^**^ |  |
|  |  | (0.014) | (0.023) | (0.019) |  |
| C disabled * V no disabled ID |  | 0.046^***^ | 0.049^**^ | 0.042^**^ |  |
|  |  | (0.011) | (0.015) | (0.016) |  |
| C blind * V disabled ID |  |  |  |  | 0.059^**^ |
|  |  |  |  |  | (0.019) |
| C blind * V no disabled ID |  |  |  |  | 0.030^*^ |
|  |  |  |  |  | (0.015) |
| C Deaf * V disabled ID |  |  |  |  | 0.063^**^ |
|  |  |  |  |  | (0.021) |
| C Deaf * V no disabled ID |  |  |  |  | 0.038^*^ |
|  |  |  |  |  | (0.015) |
| C paraplegic * V disabled ID |  |  |  |  | 0.074^***^ |
|  |  |  |  |  | (0.019) |
| C paraplegic * V no disabled ID |  |  |  |  | 0.068^***^ |
|  |  |  |  |  | (0.014) |
| Constant | 0.573^***^ | 0.578^***^ | 0.598^***^ | 0.606^***^ | 0.578^***^ |
|  | (0.014) | (0.014) | (0.020) | (0.021) | (0.015) |
| Observations | 21,601 | 21,601 | 10,935 | 10,666 | 21,601 |
| R^2^ | 0.022 | 0.024 | 0.025 | 0.012 | 0.025 |
| Adjusted R^2^ | 0.021 | 0.023 | 0.023 | 0.010 | 0.023 |

**p*<0.05, ***p*<0.01, ****p*<0.001

*Notes:* C=candidate, V=voter. Standard errors are clustered by respondent. Country and candidate fixed effects included.

**Analysis by country**

Models 3 and 4 in Table S2 show the estimates of Model 2 separately for the UK and the US. The marginal effects of candidate disability are shown in Figure S2. The estimates are very similar between the countries. The strongest difference is that the positive effect of candidate disability among citizens with a disability group identity is stronger in the UK than the US and, therefore, the difference compared to the effect among disabled citizens without the group identity is larger. Not all coefficients are statistically significant to the extent they are in the pooled sample, which is due to the smaller sample sizes of the split samples rather than smaller effect sizes.


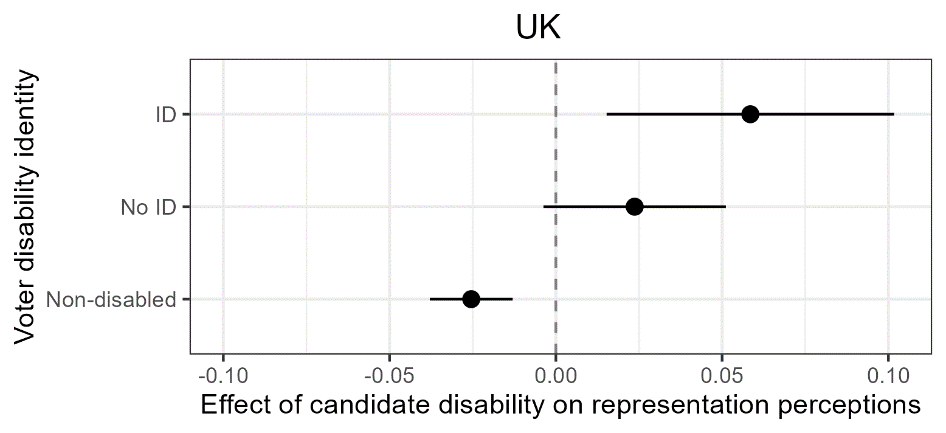


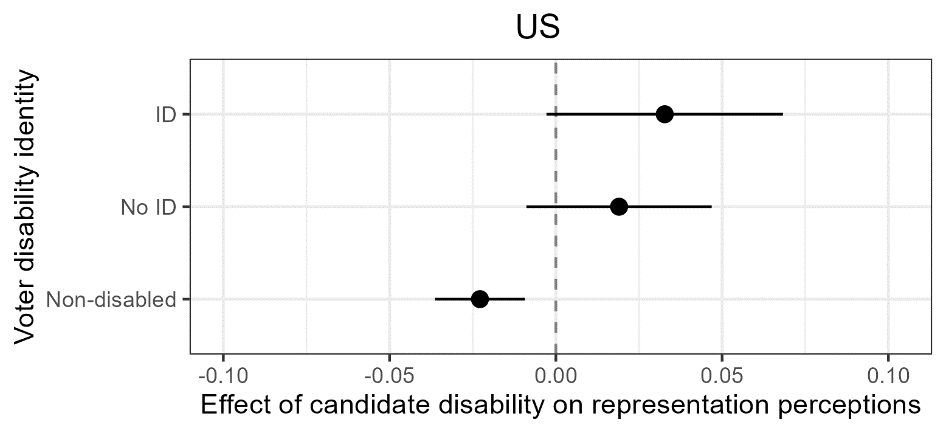


**Figure S2.** Effects of candidate disability on representation perceptions, by disability identity in UK and US

*Notes:* Full estimates in Table S2 (Models 3 and 4)

**Analysis by candidate disability type**

Model 5 in Table S2 and Figure S3 show how citizens feel represented by candidates with the three disability types. We see that all citizens feel slightly better represented by the wheelchair-using candidate than the blind or Deaf candidate. This is particularly the case for disabled citizens without a disability identity. It might be possible that disabled citizens feel particularly well represented by candidates who share their disability type – if that was the case and if a large proportion of the disabled respondents in the survey have mobility impairments, it could explain this finding. In order to explore whether this might drive the results we can split the sample of disabled respondents between those who did and those who did not indicate that they have mobility issues. Indeed, 39 per cent of disabled respondents indicated to have mobility issues (difficulty walking or climbing stairs). Yet, the findings are very similar whether we exclude respondents with or without mobility impairments, as Figure S4 shows, suggesting that they are not driven by respondents with a particular impairment type. A potential explanation might be that citizens hold lower levels of stigma about wheelchair users than Deaf and blind people. As we are not in a position to test such hypotheses here, they should be developed and tested in future research.


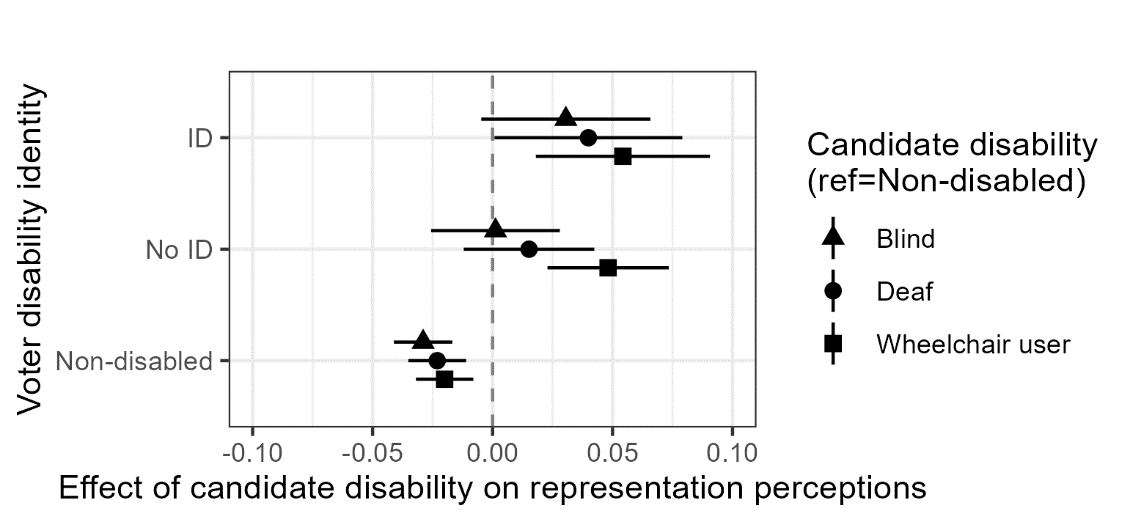


**Figure S3.** Effects of candidate disability types on representation perceptions, by disability identity

*Notes:* Full estimates in Table S2 (Model 5)


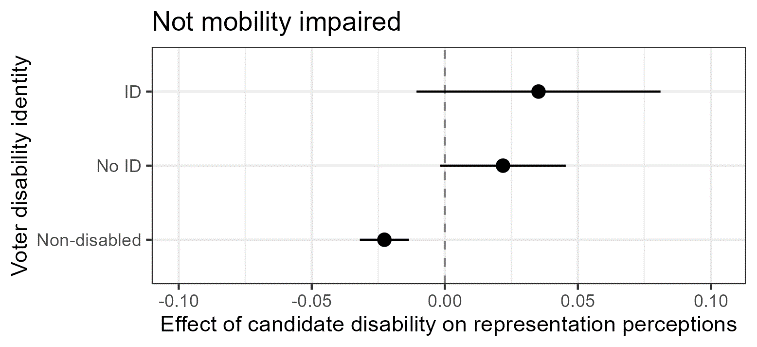

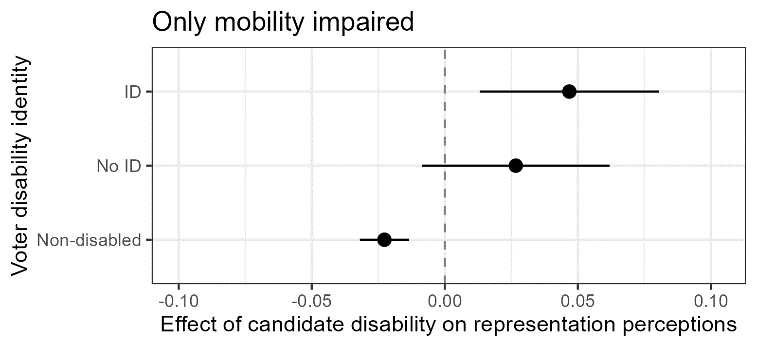


**Figure S4.** Effects of candidate disability types on representation perceptions, by disability identity, excluding respondents with and without mobility impairments

1. **Disability effects on perceived candidate preferences and citizen preferences**

**Table S3.** Linear regression of perceived candidate preferences on candidate disability

|  | Social security & welfare | | | Military & defence | Healthcare | Minority rights | Economy | Family & children | Left-right |
| --- | --- | --- | --- | --- | --- | --- | --- | --- | --- |
| C disabled | 0.037^***^ | | | -0.019^***^ | 0.045^***^ | 0.049^***^ | -0.003 | 0.008^**^ | -0.020^***^ |
|  | (0.004) | | | (0.004) | (0.003) | (0.004) | (0.003) | (0.003) | (0.004) |
| C age | 0.0003 | | | 0.0001 | -0.0001 | -0.0003 | -0.0002 | -0.0004^*^ | 0.0004^*^ |
|  | (0.0002) | | | (0.0002) | (0.0002) | (0.0002) | (0.0002) | (0.0002) | (0.0002) |
| C female | 0.007^*^ | | | -0.022^***^ | 0.009^**^ | 0.010^**^ | -0.008^*^ | 0.014^***^ | -0.008^*^ |
|  | (0.003) | | | (0.004) | (0.003) | (0.003) | (0.003) | (0.003) | (0.004) |
| C minority | 0.007 | | | -0.014^***^ | 0.002 | 0.079^***^ | -0.007^*^ | 0.001 | -0.022^***^ |
|  | (0.004) | | | (0.004) | (0.003) | (0.004) | (0.003) | (0.003) | (0.004) |
| *C profession* (ref=doctor) | | |  |  |  |  |  |  |  |
| Factory worker | 0.012^*^ | | | 0.010 | -0.055^***^ | -0.005 | 0.008 | -0.016^***^ | -0.018^**^ |
|  | (0.005) | | | (0.006) | (0.005) | (0.005) | (0.005) | (0.005) | (0.006) |
| Lawyer | -0.041^***^ | | | 0.038^***^ | -0.091^***^ | -0.025^***^ | 0.021^***^ | -0.040^***^ | 0.027^***^ |
|  | (0.006) | | | (0.006) | (0.005) | (0.006) | (0.005) | (0.005) | (0.006) |
| Small business | -0.016^**^ | | | 0.012^*^ | -0.066^***^ | -0.012^*^ | 0.038^***^ | -0.011^*^ | 0.008 |
| owner | (0.005) | | | (0.006) | (0.005) | (0.005) | (0.005) | (0.005) | (0.006) |
| Teacher | 0.007 | | | -0.009 | -0.048^***^ | 0.006 | -0.001 | 0.019^***^ | -0.018^**^ |
|  | (0.005) | | | (0.006) | (0.004) | (0.005) | (0.005) | (0.005) | (0.006) |
| C experience | 0.0002 | | | 0.0001 | 0.0003 | 0.0003 | 0.001 | 0.001 | 0.0001 |
|  | (0.0004) | | | (0.0005) | (0.0004) | (0.0004) | (0.0004) | (0.0004) | (0.0004) |
| C children | 0.006^***^ | | | 0.001 | 0.004^**^ | 0.002 | -0.0001 | 0.043^***^ | -0.001 |
|  | (0.002) | | | (0.002) | (0.001) | (0.002) | (0.001) | (0.002) | (0.002) |
| C office | 0.004 | | | 0.007 | 0.0002 | 0.004 | 0.005 | 0.004 | 0.007^*^ |
|  | (0.003) | | | (0.004) | (0.003) | (0.004) | (0.003) | (0.003) | (0.004) |
| *C party* (ref=No party) | |  | |  |  |  |  |  |  |
| Democrat/Labour | 0.028^***^ | | | -0.033^***^ | 0.013^***^ | 0.027^***^ | -0.030^***^ | 0.003 | -0.118^***^ |
|  | (0.003) | | | (0.004) | (0.003) | (0.004) | (0.003) | (0.003) | (0.003) |
| Republican/ | -0.061^***^ | | | 0.062^***^ | -0.049^***^ | -0.069^***^ | 0.021^***^ | -0.020^***^ | 0.129^***^ |
| Conservative | (0.004) | | | (0.004) | (0.004) | (0.004) | (0.003) | (0.003) | (0.004) |
| Constant | 0.734^***^ | | | 0.646^***^ | 0.882^***^ | 0.729^***^ | 0.797^***^ | 0.779^***^ | 0.517^***^ |
|  | (0.012) | | | (0.014) | (0.011) | (0.012) | (0.011) | (0.012) | (0.013) |
| Observations | 21,288 | | | 19,677 | 21,757 | 20,781 | 21,036 | 21,990 | 20,369 |
| R^2^ | 0.032 | | | 0.051 | 0.041 | 0.052 | 0.016 | 0.052 | 0.124 |
| Adjusted R^2^ | 0.032 | | | 0.050 | 0.041 | 0.051 | 0.015 | 0.051 | 0.123 |

**p*<0.05, ***p*<0.01, ****p*<0.001

*Notes:* C=candidate, V=voter. Standard errors are clustered by respondent. Country and candidate fixed effects included. Left-right ideology is coded so that low values mean left and high values right.


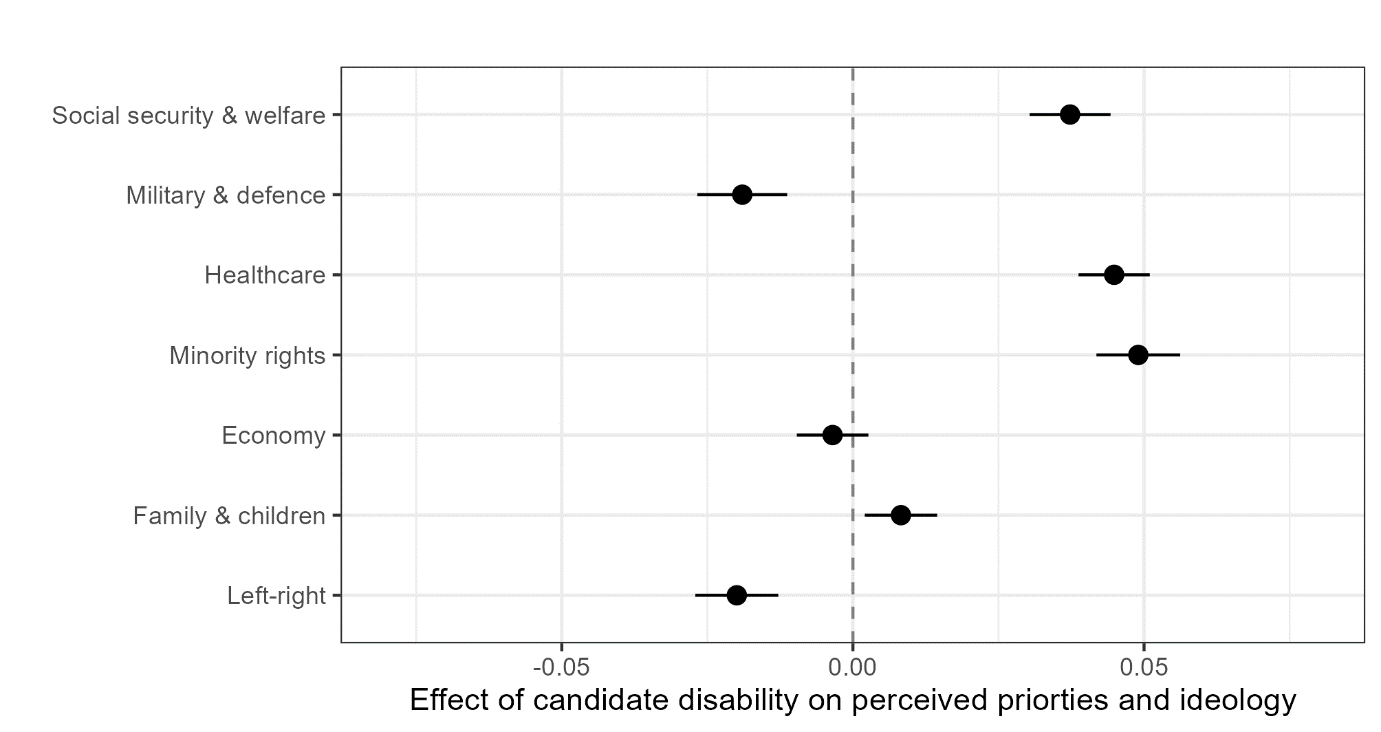


**Figure S5.** Effects of candidate disability on citizen perceptions of candidates’ issue importance

*Notes:* Full estimates in Table S3. Left-right ideology is coded so that low values mean left and high values right.

Figure S5 shows how candidate disability affects citizen perceptions of candidates’ policy priorities. Again, the coefficients are AMCEs of candidate disability from linear regressions which also include the other candidate characteristics as covariates. Positive coefficients mean that the disabled candidates are perceived as considering the issue more important than non-disabled candidates. We find this to be the case for social security and welfare, healthcare, and minority rights, as well as a small effect for family and children. Meanwhile, non-disabled candidates are perceived as considering military and defence as more important than disabled candidates (even though disabled politicians particularly in the US have often been veterans). On the left-right dimension, the negative coefficient means that disabled candidates are seen as more left-wing than non-disabled candidates. These findings reflect those in Reher (2022).

To get an indication of whether disabled citizens perceive disabled candidates as sharing their policy priorities, we regressed citizens’ issue importance on the three-category measure of disability identity (incl. non-disabled), controlling for gender and age, since women tend to be more likely to be disabled while there is a very strong positive relationship between age and disability. Figure S6 shows the coefficients for disabled citizens with and without a disability identity, with non-disabled citizens as the reference category. The results are somewhat surprising given previous findings on the effects of disability on public opinion (Gastil 2000; Schur and Adya 2013; Reher 2022). We see very few differences in policy priorities between disabled and non-disabled citizens, and rather little overlap with perceptions of candidates’ priorities. Citizens with a disability identity consider social security and welfare statistically significantly more important than non-disabled citizens (and disabled citizens with no disability identity). Meanwhile, disabled citizens are no more concerned about healthcare or minority rights. They do, however, place less emphasis on the economy than non-disabled citizens. Disabled citizens who do not identify with the disability community are statistically significantly to the right of non-disabled citizens on the ideological scale, whereas the coefficient is in the same direction but not statistically significant for those with a disability identity. This is noteworthy given that disabled people in Britain have been shown to be more left-wing (Reher 2022), while Schur and Adya (2013) found no disability gaps on the liberal-conservative scale or in Democrat and Republican support.

Comparing the disability effects between citizens and candidates, it does not look as if disabled citizens overall necessarily perceive disabled candidates to be closer to them. Yet, these figures are aggregate measures; it is still possible that individual citizens’ representation perceptions are driven by perceived issue preference congruence.

**Table S4.** Linear regression of citizen preferences on disability status and identity

|  | Social security & welfare | Military & defence | Healthcare | Minority rights | Economy | Family & children | Left-right |
| --- | --- | --- | --- | --- | --- | --- | --- |
| *V disability identity* (ref=non-disabled) |  |  |  |  |  |  |  |
| Disabled ID | 0.039^***^ | 0.016 | 0.006 | 0.015 | -0.034^***^ | -0.002 | 0.007 |
|  | (0.010) | (0.011) | (0.008) | (0.012) | (0.008) | (0.010) | (0.013) |
| No disabled ID | 0.001 | -0.007 | -0.003 | 0.001 | -0.033^***^ | -0.007 | 0.012 |
|  | (0.008) | (0.009) | (0.006) | (0.009) | (0.006) | (0.007) | (0.010) |
| V age | 0.017^***^ | 0.025^***^ | 0.015^***^ | -0.023^***^ | 0.021^***^ | 0.006^***^ | 0.009^***^ |
|  | (0.002) | (0.002) | (0.001) | (0.002) | (0.001) | (0.002) | (0.002) |
| *V gender* (ref=female) |  |  |  |  |  |  |  |
| Male | -0.038^***^ | -0.033^***^ | -0.032^***^ | -0.062^***^ | -0.008 | -0.041^***^ | 0.054^***^ |
|  | (0.006) | (0.006) | (0.004) | (0.007) | (0.005) | (0.005) | (0.007) |
| Other | -0.075 | -0.144^*^ | 0.007 | 0.045 | -0.094 | -0.108 | -0.162^*^ |
|  | (0.060) | (0.068) | (0.048) | (0.074) | (0.049) | (0.058) | (0.080) |
| Constant | 0.789^***^ | 0.712^***^ | 0.866^***^ | 0.820^***^ | 0.816^***^ | 0.849^***^ | 0.505^***^ |
|  | (0.006) | (0.007) | (0.005) | (0.007) | (0.005) | (0.006) | (0.008) |
| Observations | 5,675 | 5,675 | 5,675 | 5,675 | 5,675 | 5,675 | 5,153 |
| R^2^ | 0.025 | 0.032 | 0.025 | 0.039 | 0.042 | 0.012 | 0.016 |
| Adjusted R^2^ | 0.024 | 0.031 | 0.024 | 0.038 | 0.041 | 0.011 | 0.015 |

**p*<0.05, ***p*<0.01, ****p*<0.001

*Notes:* Left-right ideology is coded so that low values mean left and high values right.


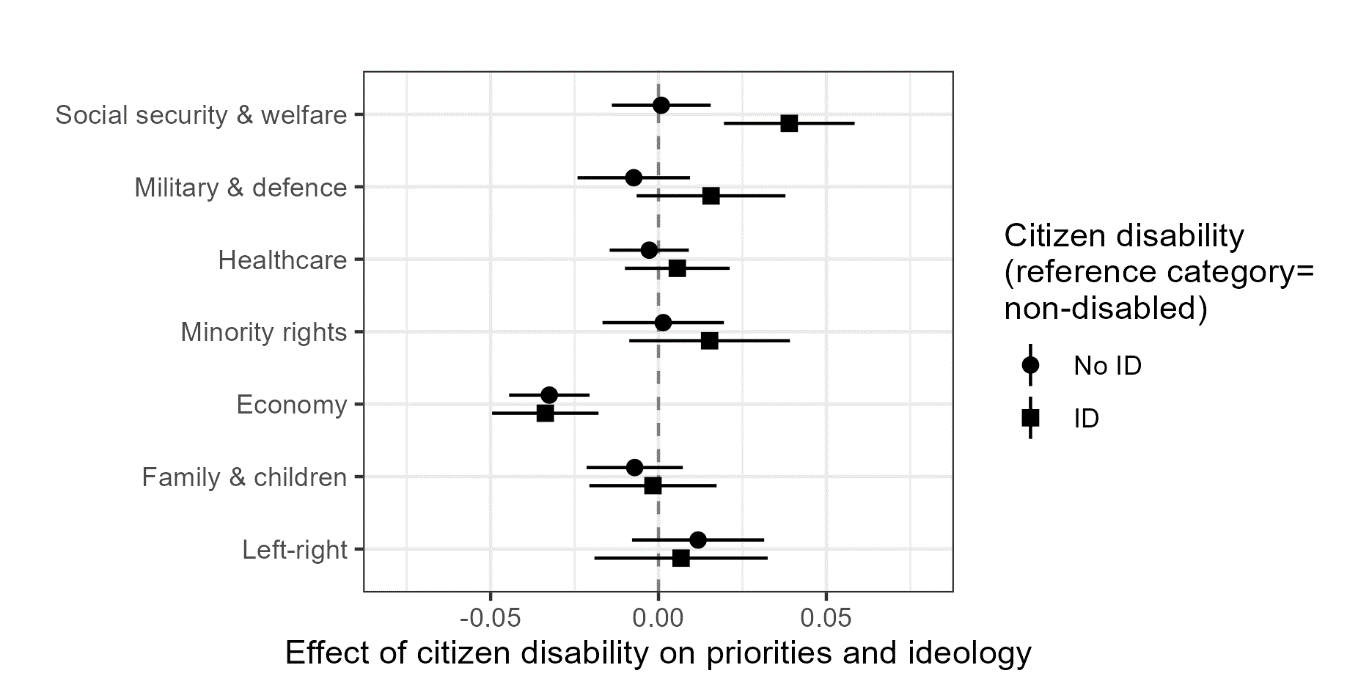


**Figure S6.** Citizens’ issue importance (based on linear regressions, controlling for age and gender)

*Notes:* Full estimates in Table S4. Left-right ideology is coded so that low values mean left and high values right.

1. **Mediation analysis**

**Table S5.** Mediation models among disabled citizens with a disability identity

|  | Representation perceptions (Base model) | Congruence perceptions (Mediator model) | Representation perceptions (Outcome model) |
| --- | --- | --- | --- |
|  | (1) | (2) | (3) |
| Congruence perceptions |  |  | 0.788^***^ |
|  |  |  | (0.058) |
| C disabled | 0.043^**^ | 0.017^**^ | 0.030^*^ |
|  | (0.014) | (0.005) | (0.014) |
| C age | 0.0003 | 0.0001 | 0.0002 |
|  | (0.001) | (0.0003) | (0.001) |
| C female | -0.007 | -0.002 | -0.005 |
|  | (0.014) | (0.005) | (0.013) |
| C minority | -0.005 | 0.010 | -0.013 |
|  | (0.015) | (0.006) | (0.015) |
| *C profession* (ref=doctor) |  |  |  |
| Factory worker | 0.036 | -0.013 | 0.046^*^ |
|  | (0.022) | (0.009) | (0.021) |
| Lawyer | -0.061^**^ | -0.021^*^ | -0.045^*^ |
|  | (0.023) | (0.009) | (0.022) |
| Small business owner | 0.025 | -0.008 | 0.031 |
|  | (0.022) | (0.009) | (0.021) |
| Teacher | 0.035 | -0.013 | 0.045^*^ |
|  | (0.022) | (0.008) | (0.021) |
| C experience | 0.002 | 0.001 | 0.001 |
|  | (0.002) | (0.001) | (0.002) |
| C children | 0.014^*^ | 0.004 | 0.011 |
|  | (0.006) | (0.002) | (0.006) |
| C office | -0.003 | 0.006 | -0.007 |
|  | (0.014) | (0.005) | (0.013) |
| *C party* (ref=No party) |  |  |  |
| Democrat/Labour | -0.017 | -0.007 | -0.011 |
|  | (0.017) | (0.007) | (0.016) |
| Republican/ Conservative | -0.068^***^ | -0.021^**^ | -0.051^**^ |
|  | (0.017) | (0.007) | (0.016) |
| Constant | 0.540^***^ | 0.690^***^ | -0.003 |
|  | (0.051) | (0.020) | (0.063) |
| Observations | 1,814 | 1,814 | 1,814 |
| R^2^ | 0.035 | 0.022 | 0.124 |
| Adjusted R^2^ | 0.026 | 0.012 | 0.115 |

**p*<0.05, ***p*<0.01, ****p*<0.001

*Notes:* C=candidate, V=voter. Standard errors are clustered by respondent. Country and candidate fixed effects included.

**Table S6.** Mediation models among disabled citizens without a disability identity

|  | Representation perceptions (Base model) | Congruence perceptions (Mediator model) | Representation perceptions (Outcome model) |
| --- | --- | --- | --- |
|  | (1) | (2) | (3) |
| Congruence perceptions |  |  | 0.851^***^ |
|  |  |  | (0.040) |
| C disabled | 0.021^*^ | 0.003 | 0.019^*^ |
|  | (0.010) | (0.004) | (0.009) |
| C age | -0.0002 | 0.0001 | -0.0003 |
|  | (0.001) | (0.0002) | (0.001) |
| C female | 0.011 | -0.002 | 0.013 |
|  | (0.010) | (0.004) | (0.009) |
| C minority | -0.018 | -0.003 | -0.016 |
|  | (0.011) | (0.004) | (0.010) |
| *C profession* (ref=doctor) |  |  |  |
| Factory worker | 0.013 | -0.017^**^ | 0.027 |
|  | (0.015) | (0.006) | (0.014) |
| Lawyer | -0.023 | -0.013^*^ | -0.012 |
|  | (0.016) | (0.006) | (0.015) |
| Small business owner | -0.009 | -0.007 | -0.003 |
|  | (0.015) | (0.006) | (0.014) |
| Teacher | 0.012 | -0.013^*^ | 0.023 |
|  | (0.015) | (0.006) | (0.015) |
| C experience | 0.002 | -0.001 | 0.002 |
|  | (0.001) | (0.0005) | (0.001) |
| C children | 0.003 | 0.002 | 0.002 |
|  | (0.004) | (0.002) | (0.004) |
| C office | 0.009 | 0.0005 | 0.009 |
|  | (0.010) | (0.004) | (0.009) |
| *C party* (ref=No party) |  |  |  |
| Democrat/Labour | 0.011 | -0.012^*^ | 0.021 |
|  | (0.012) | (0.005) | (0.011) |
| Republican/ Conservative | -0.026^*^ | -0.016^***^ | -0.012 |
|  | (0.012) | (0.005) | (0.011) |
| Constant | 0.539^***^ | 0.720^***^ | -0.074 |
|  | (0.036) | (0.014) | (0.045) |
| Observations | 3,507 | 3,507 | 3,507 |
| R^2^ | 0.028 | 0.008 | 0.140 |
| Adjusted R^2^ | 0.023 | 0.003 | 0.135 |

**p*<0.05, ***p*<0.01, ****p*<0.001

*Notes:* C=candidate, V=voter. Standard errors are clustered by respondent. Country and candidate fixed effects included.

**Table S7.** Mediation models among non-disabled citizens

|  | Representation perceptions (Base model) | Congruence perceptions (Mediator model) | Representation perceptions (Outcome model) |
| --- | --- | --- | --- |
|  | (1) | (2) | (3) |
| Congruence perceptions |  |  | 0.792^***^ |
|  |  |  | (0.019) |
| C disabled | -0.025^***^ | 0.002 | -0.027^***^ |
|  | (0.004) | (0.002) | (0.004) |
| C age | -0.0003 | -0.00003 | -0.0003 |
|  | (0.0002) | (0.0001) | (0.0002) |
| C female | 0.005 | -0.001 | 0.005 |
|  | (0.004) | (0.002) | (0.004) |
| C minority | -0.015^**^ | -0.002 | -0.014^**^ |
|  | (0.005) | (0.002) | (0.005) |
| *C profession* (ref=doctor) |  |  |  |
| Factory worker | -0.001 | -0.008^**^ | 0.005 |
|  | (0.007) | (0.003) | (0.007) |
| Lawyer | -0.053^***^ | -0.010^***^ | -0.045^***^ |
|  | (0.007) | (0.003) | (0.007) |
| Small business owner | -0.005 | -0.005 | -0.001 |
|  | (0.007) | (0.003) | (0.007) |
| Teacher | 0.009 | -0.006^*^ | 0.013^*^ |
|  | (0.007) | (0.003) | (0.007) |
| C experience | 0.001 | 0.0001 | 0.001 |
|  | (0.001) | (0.0002) | (0.001) |
| C children | 0.014^***^ | 0.005^***^ | 0.010^***^ |
|  | (0.002) | (0.001) | (0.002) |
| C office | 0.009^*^ | 0.001 | 0.009^*^ |
|  | (0.004) | (0.002) | (0.004) |
| *C party* (ref=No party) |  |  |  |
| Democrat/Labour | 0.001 | 0.015^***^ | -0.010^*^ |
|  | (0.005) | (0.002) | (0.005) |
| Republican/ Conservative | -0.036^***^ | 0.004 | -0.040^***^ |
|  | (0.006) | (0.002) | (0.006) |
| Constant | 0.587^***^ | 0.698^***^ | 0.034 |
|  | (0.016) | (0.006) | (0.020) |
| Observations | 15,988 | 15,988 | 15,988 |
| R^2^ | 0.024 | 0.008 | 0.120 |
| Adjusted R^2^ | 0.023 | 0.007 | 0.119 |

**p*<0.05, ***p*<0.01, ****p*<0.001

*Notes:* C=candidate, V=voter. Standard errors are clustered by respondent. Country and candidate fixed effects included.

**Table S8.** Mediation analysis with candidate disability as treatment, perceived congruence as mediator, and representation perceptions as outcome

|  | Disability ID | No disability ID | Non-disabled |
| --- | --- | --- | --- |
| Average Causal Mediation Effect (ACME) | 0.013**  [0.005, 0.02] | 0.003  [-0.004, 0.01] | -0.002  [-0.001, 0.00] |
| Average Direct Effect (ADE) | 0.031*  [0.003, 0.06] | 0.019*  [-0.000, 0.04] | -0.027***  [-0.035, -0.02] |
| Total Effect | 0.044**  [0.016, 0.07] | 0.022*  [0.000, 0.04] | -0.025***  [-0.033, -0.02] |
| Proportion mediated | 0.297**  [0.119, 0.76] | 0.129  [-0.357, 0.63] | -0.078  [-0.232, 0.02] |

**p*<0.05, ***p*<0.01, ****p*<0.001.

*Notes:* Estimates with Quasi-Bayesian Confidence Intervals from 1000 simulations, with robust standard errors.

A key assumption of mediation analysis is sequential ignorability, meaning there are no unobserved pre-treatment confounders which affect both the mediator and the outcome. This assumption cannot be tested with the observed date, but sensitivity analysis allows evaluating the robustness of the results to potential violation of this assumption (Imai and Yamamoto 2013; Tingley et al. 2014). The sensitivity parameter ρ is the correlation between the residuals of the mediator and the outcome regression. “If there exist unobserved pre-treatment confounders which affect both the mediator and the outcome, we expect that the sequential ignorability assumption is violated and ρ is no longer zero. The sensitivity analysis is conducted by varying the value of ρ and examining how the estimated ACME changes” (Tingley et al. 2014: 14). For all three groups, the ACME equals zero if ρ equals 0.3, which indicates that it is moderately robust to the possible unobserved pre-treatment mediator–outcome confounding, though slightly less compared to some previous studies (cf. Imai and Yamamoto 2013; Imai et al. 2011).


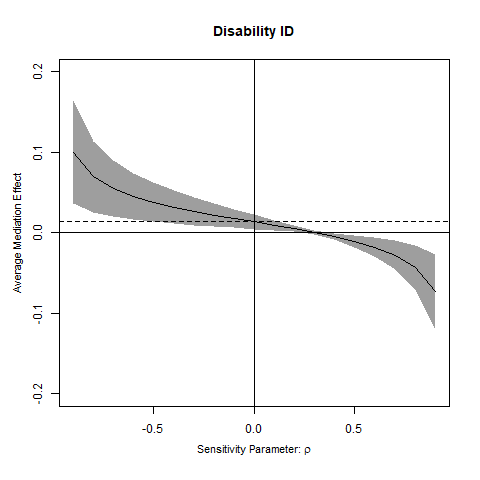

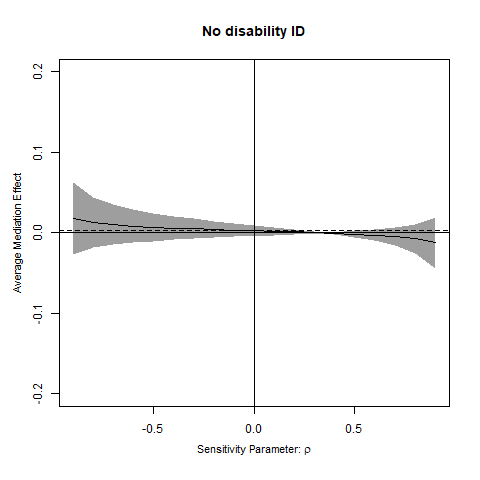

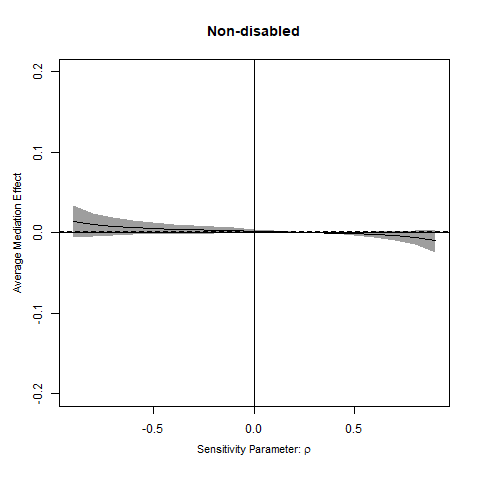


**Figure S7.** Sensitivity analysis for sequential ignorability assumption

1. **Analysis of relationships between candidates’ and respondents’ disability status and identity**

Respondents were asked after completing the experiment and answering the outcome questions about the candidates about their own sociodemographic characteristics, including whether they considered themselves disabled and – if yes – whether they identify with the disability community. While asking these questions after the experiment avoids the risks of priming respondents or helping them guess the purpose of the experiment (thus introducing demand effects and/or social desirability bias), it introduces the risk of post-treatment bias, which can occur if control or moderating variables are measured post-treatment. As Montgomery et al. (2018: 772) explain,

[…] researchers may measure a moderator after their experimental manipulation and estimate a statistical model including an interaction term. For these models to be valid, the moderator x must not be affected by the experimental randomization. […] Even variables that seem likely to remain fixed when measured after treatment, such as measures of racial or partisan identification, can be affected by treatments […].

More specifically, the risk in our study might be that disabled respondents who saw more disabled candidates (or at least one as opposed to none) felt more at ease to disclose their own disability and more encouraged to indicate that they identify with the disability community, given that they were just presented with an example, or several examples, of a disabled person who has succeeded in standing for office. This might have reduced their perception of stigma among both the researcher and society more widely, and as a result reduced their self-stigma and induced a positive sense of identity. The first counterargument would be that identifying as disabled, as well as belonging to the disability community, cannot be influenced this easily. Second, the survey is explicitly anonymous, meaning that most respondents are likely to not have worried about disclosing a disability or other characteristics for confidentiality reasons. Third, most respondents were presented at least one disabled candidate – only 2.8 per cent of the sample saw no disabled candidate.

Montgomery, Nyhan and Torres point out that “posttreatment bias cannot be easily diagnosed or remedied empirically” (2018: 772). At the same time, they do acknowledge our dilemma, asserting that “asking questions about certain highly salient covariates like group identification before an outcome variable can affect subsequent responses” and “further research is needed on how to minimize potential priming effects” (2018: 773). Thus, while we acknowledge that testing whether the distributions of the post-treatment variables differ between experimental treatment groups does not allow us to rule out post-treatment bias (Montgomery et al. 2018: 773), we nevertheless provide these analyses in order to give a sense of the potential severity of the issue. We test whether respondents’ likelihood of indicating a disability or identifying with the disability community is associated with (a) whether they saw a disabled candidate amongst the four candidate profiles and (b) how many disabled candidates they saw.

**Table S9.** Relationship between candidate disability and respondent disability status

|  |  | Respondent disability | | | Chi-squared=  0.98(1), *p*=0.323 |
| --- | --- | --- | --- | --- | --- |
|  |  | Non-disabled | Disabled | Total |  |
| Respondent saw disabled candidate(s) | No | 127 (76.0%) | 40 (23.9%) | 167 (2.9%) |  |
|  | Yes | 4131 (72.6%) | 1560 (27.4%) | 5691 (97.1%) |  |
|  | Total | 4258 | 1600 | 5858 |  |

**Table S10.** Relationship between candidate disability and respondent disability group identity

|  |  | Respondent disability group identity | | | Chi-squared=  0.71(1), *p*=0.399 |
| --- | --- | --- | --- | --- | --- |
|  |  | No identity | Identity | Total |  |
| Respondent saw disabled candidate(s) | No | 26 (72.2%) | 10 (27.8%) | 32 (2.5%) |  |
|  | Yes | 904 (65.5%) | 477 (34.5%) | 1381 (97.5%) |  |
|  | Total | 930 | 487 | 1417 |  |

Table S9 shows that respondents who saw at least one disabled candidate were slightly more likely to disclose a disability than those who saw no disabled candidate. However, these differences are not statistically significant, presumably due to the low number of respondents who saw no disabled candidates. A comparison of the mean number of disabled candidates which respondents identifying as non-disabled (0.589) and respondents identifying as disabled (0.585) saw shows that there is no substantively or statistically significant difference (*t*=0.47(2974), *p*=0.640). The same applies to disability group identity (Table 10): disabled respondents without this identity saw 0.582 disabled candidates on average while those with a group identity saw 0.592 (*t*=-0.71(966), *p*=0.450). From these results, it seems that if anything, respondents’ declared disability status and group identity are related to whether they saw one or more disabled candidates as opposed to none. This is plausible, since the marginal effect of seeing more than one disabled candidate in the setting of the experiment would likely have decreasing marginal effects. Therefore, as a robustness check we replicate the main model from the analysis (Model 2, Table S2) among the subsample of respondents who saw at least one disabled candidate (Table S11). The estimates are almost identical, suggesting that including the set of respondents who did not see any disabled candidates, and therefore are unlikely to have drawn a connection between the candidates and their own disability status, did not bias the results.

Potential remaining risks include that seeing disabled candidates might have affected responses about one’s own disability in different ways, i.e., encouraging some to disclose a disability and discouraging others, which would mean the effects cancel out. It is also conceivable that disabled respondents only felt encouraged if the candidate shared other characteristics with them, e.g. party affiliation, gender, etc. As such, these analyses certainly do not rule out the risks of post-treatment bias entirely, but they provide some indications and reassurance.

**Table S11.** Linear regression of representation perceptions on candidate disability (Model 2, Table S2) among respondents who saw at least one disabled candidate

| C disabled | -0.024^***^ |
| --- | --- |
|  | (0.005) |
| *V disability identity* (ref=non-disabled) |  |
| Disabled ID | -0.025^*^ |
|  | (0.011) |
| No disabled ID | -0.014 |
|  | (0.008) |
| C age | -0.0002 |
|  | (0.0002) |
| C female | 0.003 |
|  | (0.004) |
| C minority | -0.015^***^ |
|  | (0.004) |
| *C profession* (ref=doctor) |  |
| Factory worker | 0.004 |
|  | (0.006) |
| Lawyer | -0.048^***^ |
|  | (0.006) |
| Small business owner | -0.004 |
|  | (0.006) |
| Teacher | 0.011 |
|  | (0.006) |
| C experience | 0.001^*^ |
|  | (0.0005) |
| C children | 0.012^***^ |
|  | (0.002) |
| C office | 0.008^*^ |
|  | (0.004) |
| *C party* (ref=Democrat/Labour) |  |
| No party | -0.0005 |
|  | (0.004) |
| Republican/Conservative | -0.037^***^ |
|  | (0.005) |
| *Interaction C disability * V disability identity* (ref=non-disabled) |  |
| C disabled * V disabled ID | 0.066^***^ |
|  | (0.015) |
| C disabled * V no disabled ID | 0.043^***^ |
|  | (0.011) |
| Constant | 0.578^***^ |
|  | (0.015) |
| Observations | 20,980 |
| R^2^ | 0.024 |
| Adjusted R^2^ | 0.023 |

**p*<0.05, ***p*<0.01, ****p*<0.001

*Notes:* C=candidate, V=voter. Standard errors are clustered by respondent. Country and candidate fixed effects included.

**References**

Gastil, J. 2000. ‘The Political Beliefs and Orientations of People with Disabilities.’ *Social Science Quarterly* 81: 588-603.

Imai, K., L. Keele, and D. Tingley. 2010. ‘A General Approach to Causal Mediation Analysis.’ *Psychological Methods* 15(4): 309–334.

Imai, K., and T. Yamamoto. 2013. ‘Identification and Sensitivity Analysis for Multiple Causal Mechanisms: Revisiting Evidence from Framing Experiments.’ *Political Analysis* 21: 141–171.

Montgomery, J.M., B. Nyhan, and M. Torres. 2018. ‘How Conditioning on Posttreatment Variables Can Ruin Your Experiment and What to Do about It.’ *American Journal of Political Science* 62(3): 760-775.

Reher, S. 2022. ‘Do Disabled Candidates Represent Disabled Citizens?’ *British Journal of Political Science*. 52(2): 520-534.

Schur, L., and M. Adya. 2013. ‘Sidelined or Mainstreamed? Political Participation and Attitudes of People with Disabilities in the United States.’ *Social Science Quarterly* 94(3): 811-839.

Tingley, D., T. Yamamoto, K. Hirose, L. Keele, and K. Imai. 2014. ‘mediation: R Package for Causal Mediation Analysis.’ *Journal of Statistical Software* 59(5):1–38.
